# AIP, fatty liver, and HbA1c as modifiers of the C-index and diabetes risk relationship

**DOI:** 10.1186/s12944-025-02546-1

**Published:** 2025-04-02

**Authors:** Yanmei Liu, Rui Shi, Huiying Cao, Jian Zhang, Shuangyue Li, Xilin Kang, Yongjuan Ma, Yudian Wu, Yangfan Guo, Lei Feng

**Affiliations:** 1 Department of Clinical Laboratory, Yan’an Hospital Affiliated to Kunming Medical University, Yunnan, Email, 650000 People’s Republic of China; 2 Department of Laboratory Medicine, Sixth School of Clinical Medicine, the Affiliated Qingyuan Hospital (Qingyuan People’s Hospital), Guangzhou Medical University, Guangzhou, China; 3 Department of Clinical Laboratory, The Sixth Affiliated Hospital of Kunming Medical University, Yuxi, 650000 China; 4 Department of Clinical Laboratory, The First People’s Hospital of Honghe State, Yunnan, China; 5 Central Laboratory, Yan’an Hospital Affiliated to Kunming Medical University, Yunnan, 650000 China; 6 Yunnan Key Laboratory of Tumor Immunological Prevention and Control, Yan’an Hospital, Kunming Medical University, Yunnan, China

**Keywords:** Diabetes, Atherogenic index of plasma, Conicity index, Mediation analysis

## Abstract

**Background and aims:**

Recent studies have suggested an interplay between conicity index (C-index)-related diabetes risk and lipid burden. It is plausible that the atherogenic index of plasma (AIP), fatty liver, and HbA1c mediate the association between C-index and diabetes risk, though this has not been fully explored. This study explored whether AIP, fatty liver, and HbA1c mediate the relationship between C-index and diabetes risk, as well as their combined effect.

**Methods:**

Data from 15,453 participants in the NAGALA Cohort were analyzed (median follow-up 5.39 years). Restricted Cubic Spline (RCS) and univariate Cox regression models adjusted for risk factors were used to assess the role of AIP in modifying the C-index-diabetes relationship. Mediation analysis assessed the contributing factors, and predictive models for diabetes were established.

**Results:**

Among normoglycemic individuals, the AIP and C-index remained significantly and positively associated with diabetes risk. Higher AIP levels strengthened the C-index-diabetes association, particularly in the AIP range of 0.11-≤1.21. In the initial model, hazard ratios (HRs) for those in the fourth quartile of the C-index distribution in this group showed a significant HR of 2.22 (1.37–3.59). As fatty liver and HbA1c levels were progressively adjusted, the HRs gradually decreased, but a significant HR of 1.70 (1.05–2.76) was retained in the fully adjusted model. No significant association was observed in the other AIP strata. Furthermore, AIP, fatty liver, and HbA1c mediated the relationship between C-index and diabetes risk, with mediation effects of 9.8%, 25.0%, and 13.4%, respectively. Notably, the combined model incorporating AIP, fatty liver, HbA1c, and the C-index achieved the highest predictive performance (AUC = 0.86), outperforming the C-index alone (AUC = 0.68).

**Conclusions:**

C-index was significantly associated with diabetes risk, modified by AIP, fatty liver, and HbA1c. These findings emphas ize the importance of AIP along with the C-index, particularly in the context of fatty liver and HbA1c, for diabetes risk screening and management.

**Supplementary Information:**

The online version contains supplementary material available at 10.1186/s12944-025-02546-1.

## Introduction

Diabetes Mellitus (DM) is a prevalent metabolic disorder affecting approximately 10.5% of the global population, with projections to rise to 783 million by 2045, posing a significant public health burden [1, 2]. The increasing prevalence of obesity as a major driver of type 2 diabetes mellitus (T2DM) exacerbates this challenge [3]. Traditionally, anthropometric measures such as Body Mass Index (BMI) and Waist Circumference (WC) have been used to assess obesity-related risks [4]. However, BMI fails to differentiate between fat and lean mass or to reflect fat distribution accurately [5], and WC, being highly correlated with BMI, has its own limitations as an independent measure of obesity [6]. These shortcomings highlight the need for more refined metrics to assess obesity and its metabolic consequences. The conicity index (C-index) is a promising new measure that assesses abdominal fat accumulation in both obese and lean individuals [7]. Notably, compared with BMI and WC, the C-index has shown stronger associations with cardiovascular diseases [8], glucose changes [9], and blood pressure [10], suggesting its potential as a more robust indicator of diabetes risk.

In parallel, atherogenic index of plasma (AIP), defined as the logarithmic transformation of the ratio of triglycerides (TG) to high-density lipoprotein cholesterol (HDL-C), has emerged as a reliable marker for evaluating obesity-related diabetes risk [11]. By accounting for both TG and HDL-C levels, AIP offers a more precise assessment of dyslipidemia’s pathogenicity compared to individual lipid markers. High TG levels promote lipotoxicity and insulin resistance [12], while HDL-C exerts protective effects on metabolic health [13]. Initially conceptualized as a cardiovascular risk marker, AIP has since been strongly linked to insulin resistance-related conditions such as obesity, prediabetes, diabetes, and metabolic syndrome [11]. Thus, exploring the relationship between AIP and diabetes risk not only deepens our understanding of diabetes pathogenesis but also informs clinical management strategies.

Fatty Liver, also referred to as Non-Alcoholic Fatty Liver Disease (NAFLD), is another critical metabolic condition characterized by liver fat accumulation independent of alcohol consumption. Fatty Liver is strongly associated with obesity, insulin resistance, and metabolic syndrome and plays a central role in the development of T2DM. Studies have shown that Fatty Liver increases diabetes risk, and the degree of liver fat accumulation correlates with insulin resistance and glucose dysregulation [14]. Additionally, glycated hemoglobin (HbA1c), a crucial biomarker for diabetes diagnosis and monitoring, reflects long-term glucose levels and is linked to the progression of diabetes [15]. Together, fatty Liver and HbA1c are pivotal in the pathogenesis of diabetes and may mediate the relationship between emerging biomarkers such as the C-index and diabetes risk.

Although previous studies have identified significant correlations between the C-index and AIP in certain populations, such as those with kidney failure [16], these associations appear weaker in the context of fatty liver disease [17]. It remains unclear whether this association exists in normoglycemic individuals, highlighting the need for further research to explore these relationships in a broader, healthier population. We hypothesize that the C-index disrupts lipid metabolism, raises AIP levels, promotes fatty liver, induces insulin resistance, and increases HbA1c. To test this hypothesis, we utilized data from 15,453 normoglycemic participants in the Japanese NAGALA cohort. The study aimed to examine the mediating roles of AIP, fatty liver, and HbA1c in the relationship between C-index and diabetes risk, providing a comprehensive understanding of their complex interactions and proposing novel strategies for diabetes risk management.

## Methods

### Data source and study population

This study utilized the NAGALA cohort (spanning 1994–2016), which was established by the Okamura team [18]. In accordance with the Dryad data-sharing policy, the dataset was publicly accessible through the Dryad repository (https://datadryad.org/stash/dataset/doi:10.5061/dryad.8q0p192). Ethical approval for the NAGALA study was granted by the Murakami Memorial Hospital and informed consent was obtained from all participants. We are currently conducting a secondary analysis of this cohort. The need for ethical approval and informed consent was waived.

### Study design and participants

**Fig. 1:**
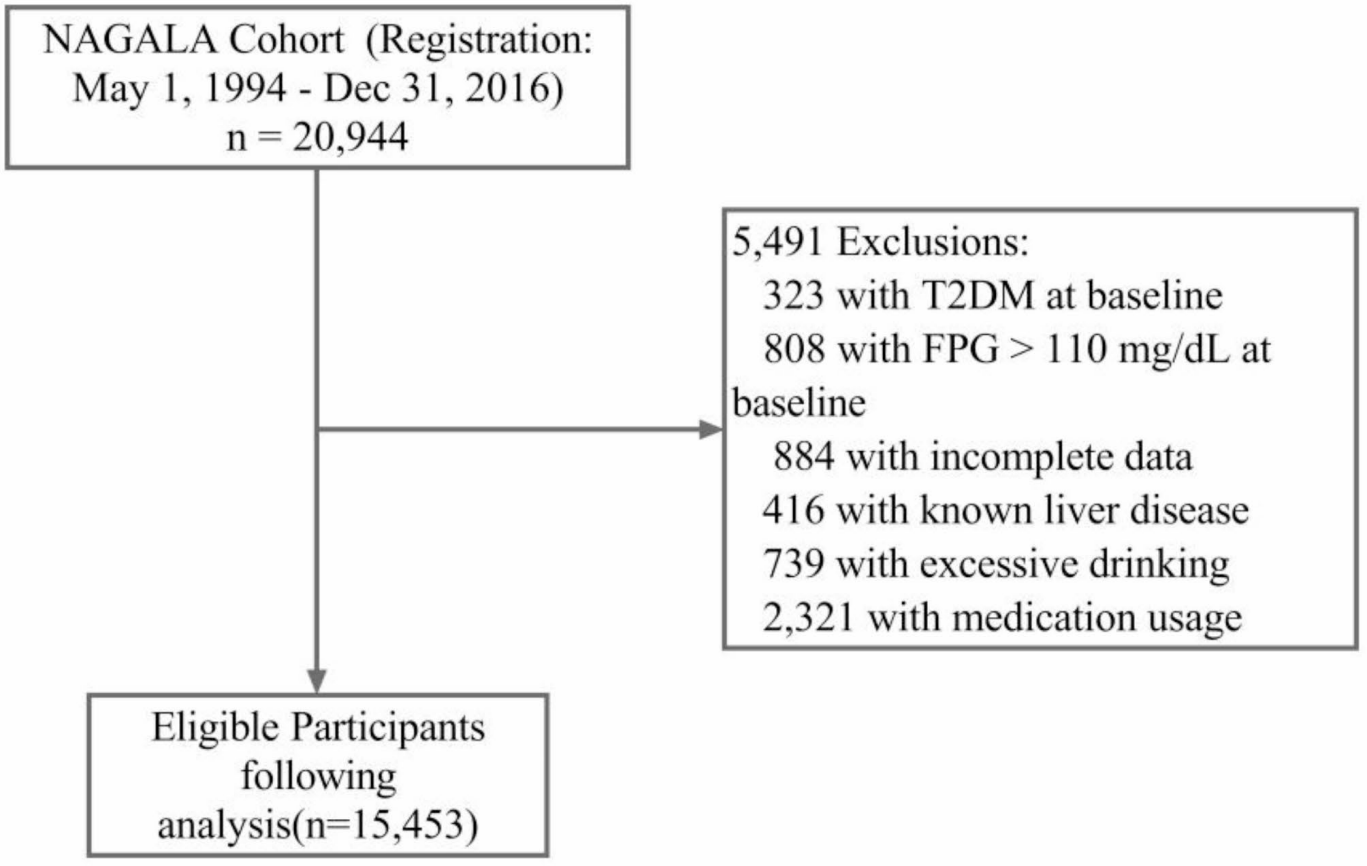
Flow chart of study participants

A total of 20,944 participants were initially included in the NAGALA cohort to investigate the effect of obesity phenotype on the risk of developing T2D. Since most of the participants require repeated examinations, the researchers conducted a follow-up study of incident T2D diagnosed by blood tests and fatty liver diagnosed by abdominal ultrasound. The inclusion criteria for the NAGALA study were described in detail in the original article [15]. A total of 15,464 participants were selected according to the following exclusion criteria (Fig. 1): (1) baseline T2DM (*n* = 323), (2) liver disease (*n* = 416), (3) baseline FPG > 110 mg/dL (*n* = 808), (4) excessive alcohol consumption (*n* = 739), (5) medication use (*n* = 2,321), and (6) incomplete data (included because of missing data of covariates, including alcohol consumption, exercise, abdominal ultrasonography or heigh) (*n* = 873). To elucidate the role of AIP, fatty liver, and HbA1c in C-index-related diabetes risk, this study was a secondary analysis of the open data from the NAGALA study. Eleven samples were excluded due to missing HDL values. Finally, 15,453 individuals completed the study.

### Data collection and measurement

Systolic blood pressure (SP), diastolic blood pressure (DP), weight, height, and WC were measured by medical personnel using standardized procedures, and age and sex were recorded via questionnaires. Blood samples were obtained after an 8-hour fasting period and analyzed for fasting plasma glucose (FPG), glycated hemoglobin (HbA1c), total cholesterol (TC), TG, HDL-C, low-density lipoprotein cholesterol (LDL-C), alanine aminotransferase (ALT), aspartate aminotransferase (AST), and gamma-glutamyl transferase (GGT). A fatty liver was diagnosed using abdominal ultrasound [19].

Lifestyle factors were categorized based on standard guidelines: alcohol consumption as nondrinking/low, mild, moderate, or heavy, with excessive drinking defined as weekly ethanol intake exceeding 60 g for men or 40 g for women [20]; smoking status as nonsmoker, former smoker, or current smoker; and regular exercise as physical activity occurring at least once per week. Baseline data were used to calculate the AIP [21] and C-index [22].

AIP was calculated with the following formula:

$$\:\text{A}\text{I}\text{P}=\text{l}\text{g}(\text{T}\text{G}/\text{H}\text{D}\text{L}-\text{C})^{21}$$

C-index was calculated with the following formula:

$$\:C-index={0.109}^{-1}WC\left(m\right){\left[\frac{weight\left(kg\right)}{height\left(m\right)}\right]}^{-1/2}5$$

### Diabetes diagnosis

Diabetes was confirmed through an HbA1c level ≥ 6.5%, an FPG concentration ≥ 126 mg/dL, or self-reporting [23]. Participants with an FPG concentration < 110 mg/dL were considered normoglycemic.

### Statistical analysis

C-index was categorized into quartiles using cohort-specific cut-points (Q), with mean cut-point values of 1.11, 1.16, and 1.21 for the normoglycemia population. The AIP group was stratified into three groups based on the relevant literature and Restricted Cubic Spline (RCS) Analysis results [24]. Participants were assigned to the AIP group at baseline. Skewed data were log-transformed.

Continuous variables are expressed as mean ± standard deviation (x ± s) for normally distributed data, while categorical variables are presented as frequencies (%). Baseline differences in continuous variables among AIP groups were assessed using one-way analysis of variance (ANOVA), whereas categorical variables were compared using the chi-square test with Bonferroni correction for multiple comparisons. Collinearity was evaluated by calculating the variance inflation factor (VIF) for each covariate using the linear regression equations. Variables with a VIF > 5 were designated as collinear [25]. The collinearity screening revealed significant collinear relationships between AIP and both TC and LDL-C, the C-index and WC, and SP and DP. Consequently, these variables (TC, LDL-C, WC, and DP) were excluded from subsequent analysis.

Multivariable Cox regression models were used to investigate the associations between AIP, C-index, and diabetes risk with incremental levels of adjustment. Model 1 was adjusted for basic demographics (age and sex), systolic blood pressure, lifestyle factors (smoking status, drinking status, and exercise habits), and liver function markers (GGT, AST, and ALT levels). Model 2 further incorporated fatty liver disease into the model. Model 3, the fully adjusted model, additionally accounted for the HbA1c levels. To assess the independent effect of the C-index on diabetes risk and its interaction with AIP, we stratified the analysis by AIP level and progressively adjusted for confounding factors using a stepwise adjustment of variables from Models 1 to 3. Furthermore, spline regression analysis was conducted to explore the relationship between the continuous C-index and future diabetes risk across different AIP values. Both biomarkers were appropriately transformed (AIP: log-transformed; C-index: scan-transformed) to meet analytical requirements. The results are reported as hazard ratios (HRs) with corresponding 95% CI.

Additionally, using Baron and Kenny’s classic regression model a mediation analysis was conducted to quantify both the direct and indirect effects of the C-index on diabetes risk through AIP, fatty liver, and HbA1c, utilizing 1000 bootstrap samples to estimate the mediation proportion. Predictive models that included the C-index, AIP, fatty liver, and HbA1c levels were assessed using AUC analysis, and DeLong tests were employed to compare the AUCs between different models. The data were divided into 70/30 training and testing sets for the performance evaluation. All statistical analyses were carried out using R (version 4.3.3) with the significance level set at *p* < 0.05.

## Results

### Characteristics of study participants

15,453 individuals from the normoglycemia group completed the study, with a mean age of 43.7 ± 8.9 years. Over a follow-up period of 5.39 years (range: 0.45–12.96 years), 373 participants were diagnosed with diabetes.

**Fig. 2:**
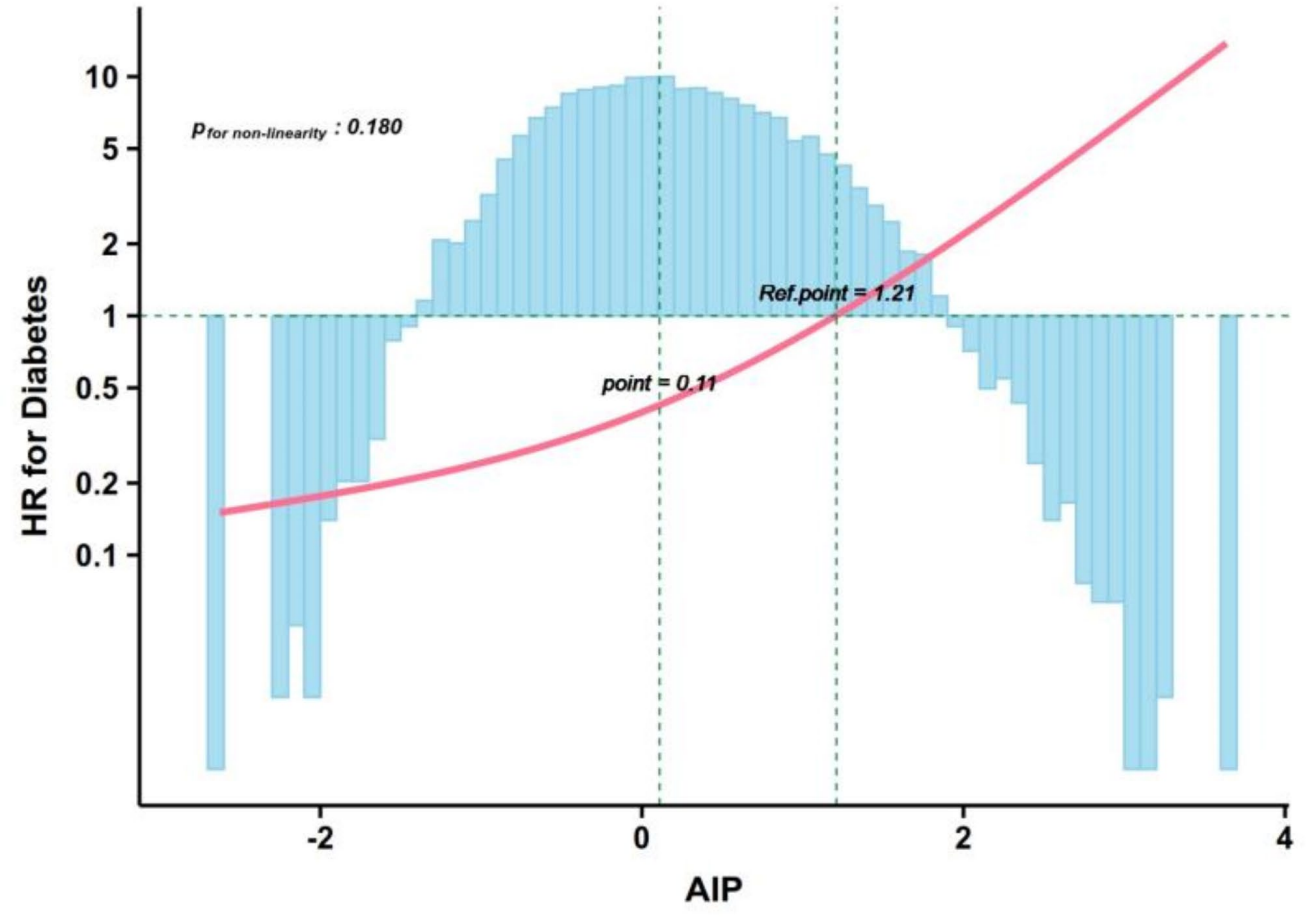
Restricted cubic spline analysis for the relationship between continuous AIP and future diabetes risk. Adjusted for age, sex, systolic pressure, drinking status, smoking status, fatty liver, exercise habits, GGT, AST, ALT, C-index, HbA1c

Furthermore, RCS demonstrated a linear dose-response relationship between AIP and diabetes risk, with AIP positively associated with diabetes risk (Fig. 2). The risk significantly increased beyond the reference point of 1.21, whereas it remained below 0.11 [24], with no significant nonlinear relationship (*p* = 0.171). Based on the literature and these findings, AIP was categorized into three groups: AIP < 0.11, 0.11 ≤ AIP ≤ 1.21, and AIP > 1.21.^24^ Significant differences were found among the three AIP groups in age, sex, BMI, waist circumference, C-index, systolic pressure, diastolic pressure, drinking status, smoking status, exercise habits, fatty liver, FPG, HbA1c, ALT, AST, GGT, TG, TC, LDL-C, and HDL-C (*p*_*adj*_ < 0.05) (Table 1).

**Table 1:** Baseline demographic and clinical characteristics of study participants

Characteristic	AIP < 0.11	0.11 ≤ AIP ≤ 1.21	AIP > 1.21	*p*-value
N	7406	6373	1674	
**Incident Diabetes**				
Non-Diabetes	7342 (99.14%)	6199 (97.27%)	1539 (91.94%)	< 0.001
Diabetes	64 (0.86%)	174 (2.73%)	135 (8.06%)	
**Follow years**	5.03 (2.22-9.00)	5.95 (2.93–9.95)	6.00 (2.93–10.03)	-
**Age**	41.00 (36.00–48.00)	44.00 (38.00–52.00)	44.00 (39.00–52.00)	< 0.001
**Sex**				
Female	4971 (67.12%)	1904 (29.88%)	159 (9.50%)	< 0.001
Male	2435 (32.88%)	4469 (70.12%)	1515 (90.50%)	
**BMI**	20.51 (19.02–22.23)	22.71 (20.92–24.67)	24.52 (22.84–26.50)	< 0.001
**WC**	0.71 (0.67–0.77)	0.79 (0.74–0.84)	0.84 (0.80–0.90)	< 0.001
**C-index**	1.14 (1.09–1.18)	1.18 (1.14–1.22)	1.20 (1.17–1.24)	< 0.001
**SP**	109.00 (100.50-118.50)	116.50 (107.50-126.50)	121.50 (112.00-131.88)	< 0.001
**DP**	67.50 (61.50–74.00)	73.00 (67.00-80.50)	77.00 (70.50–84.00)	< 0.001
**Drinking**				
no or little	6027 (81.38%)	4627 (72.60%)	1148 (68.58%)	< 0.001
light	713 (9.63%)	828 (12.99%)	213 (12.72%)	
moderate	511 (6.90%)	643 (10.09%)	203 (12.13%)	
heavy	155 (2.09%)	275 (4.32%)	110 (6.57%)	
**Smoking**				
non	5390 (72.78%)	3077 (48.28%)	560 (33.45%)	< 0.001
former	1073 (14.49%)	1466 (23.00%)	410 (24.49%)	
current	943 (12.73%)	1830 (28.71%)	704 (42.05%)	
**exercise**				
No	6037 (81.51%)	5268 (82.66%)	1442 (86.14%)	< 0.001
Yes	1369 (18.49%)	1105 (17.34%)	232 (13.86%)	
**fatty liver**				
No	7084 (95.65%)	4854 (76.17%)	778 (46.48%)	< 0.001
Yes	322 (4.35%)	1519 (23.83%)	896 (53.52%)	
**ALT**	14.00 (11.00–18.00)	19.00 (14.00–25.00)	25.00 (19.00–36.00)	< 0.001
**AST**	16.00 (13.00–20.00)	18.00 (14.00–21.00)	20.00 (16.00–25.00)	< 0.001
**GGT**	13.00 (10.00–17.00)	17.00 (13.00–25.00)	25.00 (18.00–38.00)	< 0.001
**TG**	43.00 (33.00–54.00)	88.00 (72.00-108.00)	180.50 (152.00-225.00)	< 0.001
**TC**	188.00 (169.00-211.00)	201.00 (180.00-223.00)	213.00 (191.00-237.00)	< 0.001
**LDL-C**	105.69 (90.00-122.86)	126.90 (109.31-145.01)	137.66 (118.69-156.29)	< 0.001
**HDL-C**	64.80 (56.60–74.30)	49.00 (42.90–55.70)	37.70 (33.20–42.70)	< 0.001
**FPG**	90.00 (86.00–95.00)	94.00 (90.00-100.00)	97.00 (92.00-102.00)	< 0.001
**HbA1c**	5.10 (4.90–5.40)	5.20 (5.00-5.40)	5.20 (5.00-5.50)	< 0.001

### Relationship between C-index, AIP, and diabetes risk

**Fig. 3:**
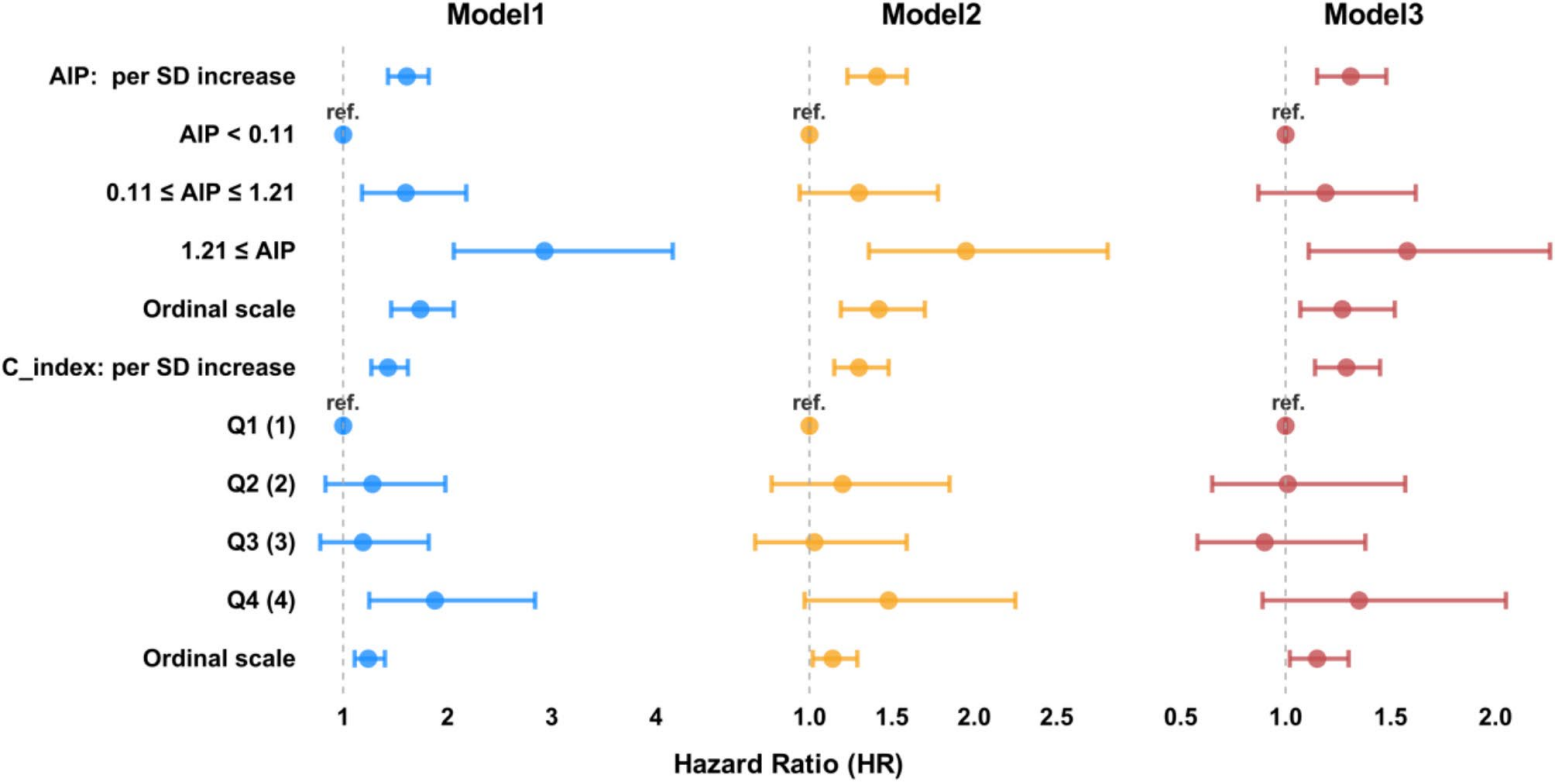
Association between AIP or C-index and risk of diabetes Models: Model 1 adjusted for age, sex, systolic pressure, exercise habits, smoking status, drinking status, ALT, AST, GGT, and AIP (solely in C-index analysis); Model 2 additionally adjusted for fatty liver; Model 3 additionally adjusted for HbA1c

When analyzed independently, elevated C-index and AIP levels were associated with the subsequent onset (Fig. 3, Supplementary Table S1). In Model 1, the C-index was significantly associated with diabetes risk, with the risk increasing progressively across the C-index quartiles, reaching its highest in the fourth quartile (HR 1.88, 95% CI: 1.25–2.84, *p* = 0.002). However, in models 2 (which additionally incorporated fatty liver) and 3 (which further incorporated HbA1c), the association between the C-index and diabetes risk was no longer significant. Similarly, AIP (≥ 0.11- < 1.21 and ≥ 1.21 vs. < 0.11) was positively associated with diabetes risk in both Models 1 and 2. Specifically, in Model 1, an AIP range of ≥ 0.11- < 1.21 was associated with an approximately 1.6-fold increased risk [HR 1.60, 95% CI: 1.18–2.18, *p* = 0.003], while AIP (≥ 1.21) was associated with a more than two-fold increased risk [HR 2.93, 95% CI: 2.06–4.16, *p* < 0.001]. In Model 2, the association between the AIP (≥ 0.11–< 1.21) and diabetes risk was no longer significant [HR 1.30, 95% CI: 0.94–1.78, *p* = 0.108]. However, for AIP (≥ 1.21), the association was attenuated, but remained significant [HR 1.95, 95% CI: 1.36–2.81), *p* < 0.001]. In fully adjusted Model 3, an elevated AIP (≥ 1.21) remained significantly associated with a 27% higher risk of diabetes [HR 1.27, 95% CI: 1.07–1.52, *p* = 0.006].

### C-index and diabetes risk across AIP strata

**Fig. 4:**
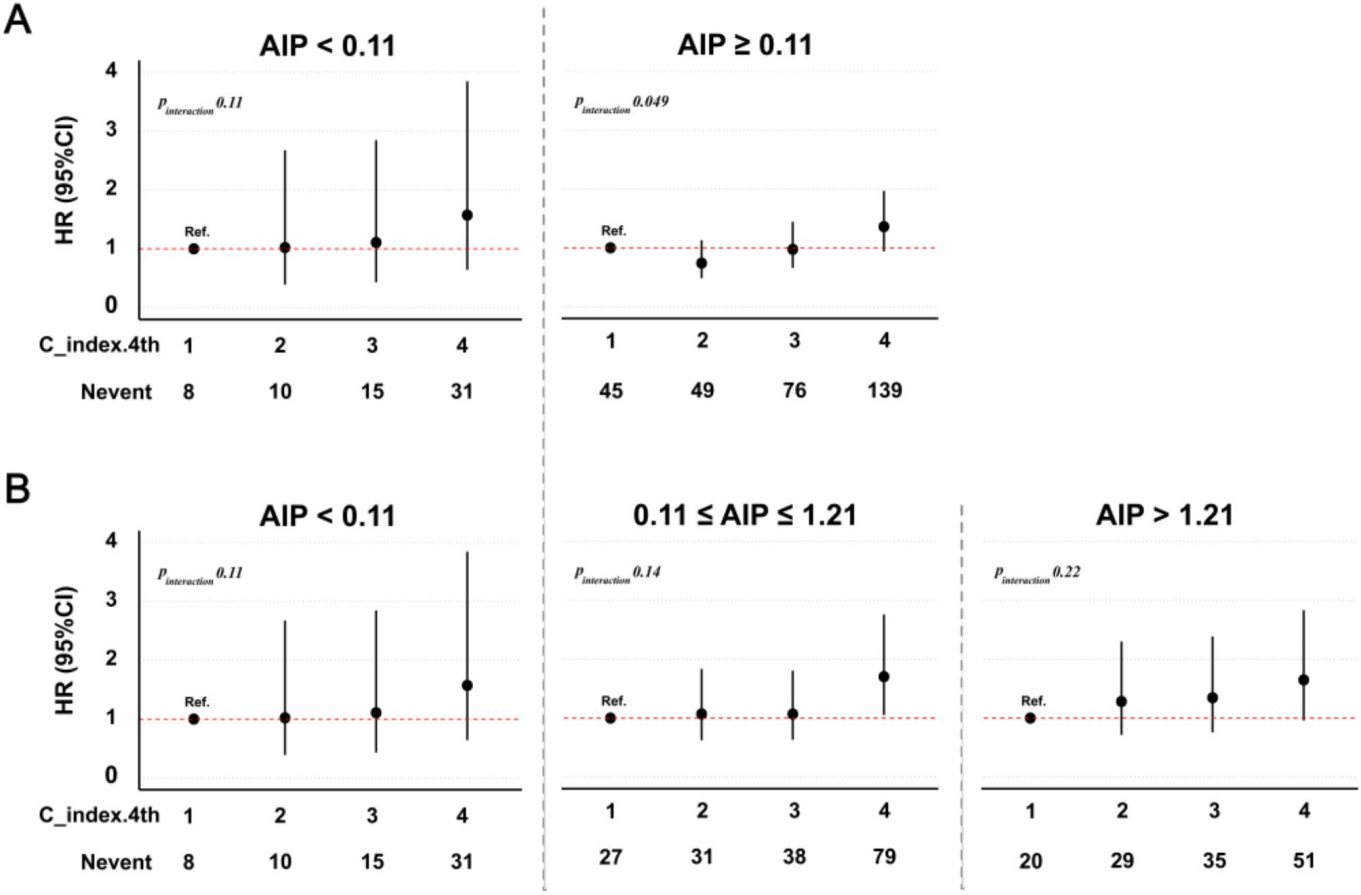
Risk of diabetes according to C-index and AIP index. (**A**) AIP index categorized into two groups: <0.11 and ≥ 0.11. (**B**) AIP index categorized into three groups: <0.11, ≥ 0.11–<1.21, and ≥ 1.21. risk and the data are presented as HRs with their 95% CI. Ref. = reference. Fully adjusted model [adjusted sex, age, systolic pressure, smoking status, drinking status, exercise habits, ALT, AST, GGT, fatty liver, and HbA1c]

We investigated whether the baseline AIP index might modify the association between C-index levels and future diabetes events. As a continuous variable, AIP significantly increased the risk of diabetes in the models (HR > 1, *p* < 0.05), with the risk increasing as the AIP levels increased (Supplementary Table S2). Assuming that AIP values ≥ 0.11 reflect a high residual lipotoxicity risk, the study population was first divided according to an AIP index of < 0.11 vs. ≥0.11 (Fig. 4A, Supplementary Table S2). Within these AIP strata, the effect of the C-index on diabetes risk was dependent on the AIP levels. For an AIP of < 0.11, the fourth C-index group did not show a significant increase in the risk of diabetes. In contrast, in the AIP ≥ 0.11 group, the fourth C-index quartile exhibited a significant effect in Model 1 (HR = 1.82, 95% CI: 1.26–2.64, *p* = 0.002), but this association was not significant in Models 2 and 3. Notably, the interaction between the AIP and C-index was significant in this group (*p*_interaction_ = 0.049), indicating the potential modifying role of AIP.

For further investigation, the AIP ≥ 0.11 group was subdivided into two categories: AIP < 1.21 and AIP ≥ 1.21 (Fig. 4B, Supplementary Table S2). Across these refined strata, the dependency of the C-index on AIP became more apparent. For an AIP < 0.11, the fourth C-index quartile did not significantly increase the risk of diabetes, which is consistent with previous findings. For AIP 0.11-≤1.21, the fourth C-index quartile significantly increased the risk of diabetes across all three models, although the effect was progressively attenuated with additional adjustments: Model 1 (HR = 2.22, 95% CI: 1.37–3.59, *p* = 0.001), Model 2 (HR = 1.65, 95% CI: 1.008-2.70, *p* = 0.046), and Model 3 (HR = 1.70, 95% CI: 1.05–2.76, *p* = 0.03). For AIP > 1.21, the fourth C-index quartile did not significantly increase the diabetes risk in any of the three models. These findings suggest that the independent effect of the C-index weakens as more control variables are added, potentially due to confounding factors (fatty liver and HbA1c).

**Fig. 5:**
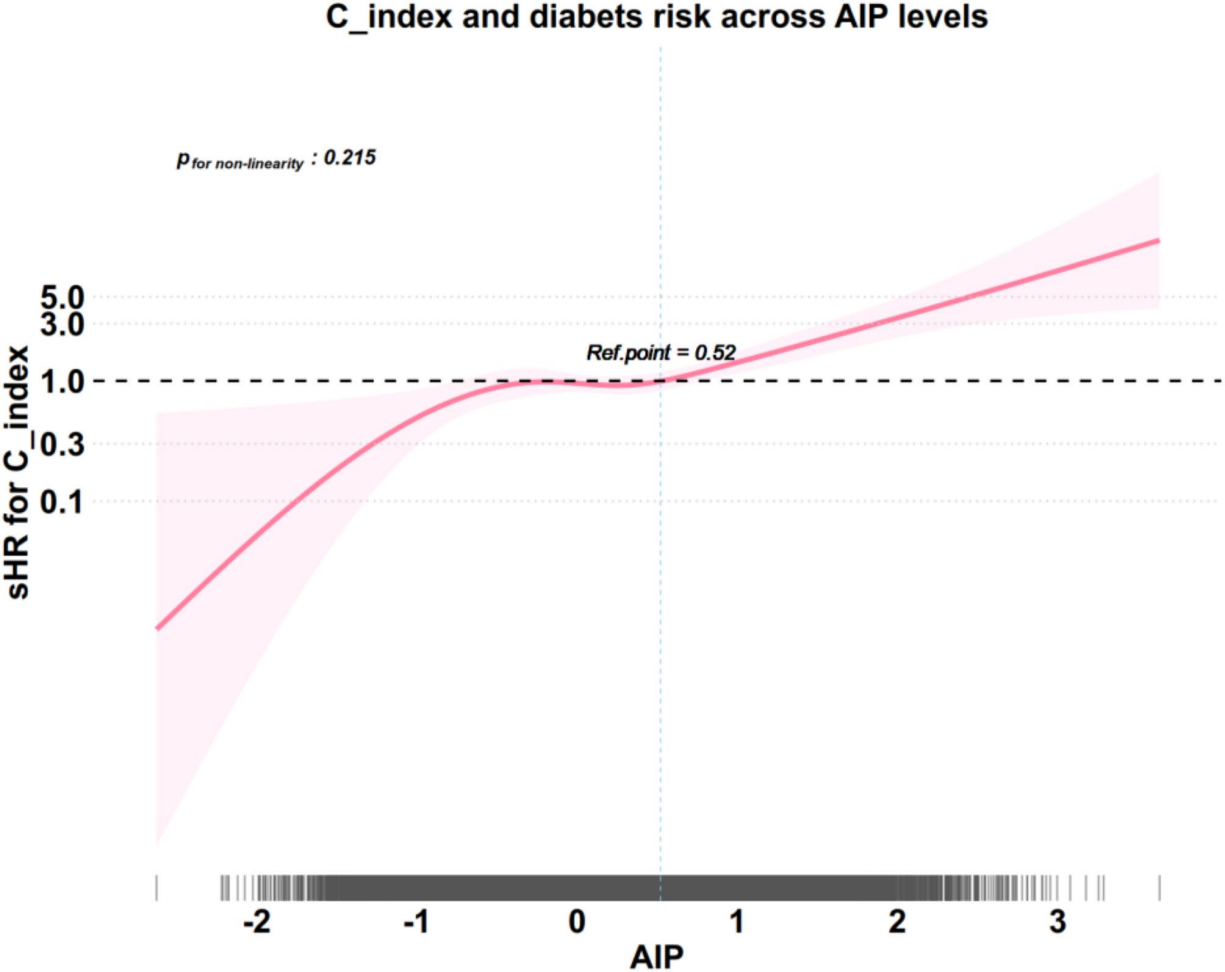
Restricted cubic spline analysis for the relationship between C-index and diabetes risk across AIP values. Fully adjusted model [adjusted sex, age, systolic pressure, smoking status, drinking status, exercise habits, ALT, AST, GGT, fatty liver, and HbA1c]

Further insights into these trends were provided by the RCS analysis (Fig. 5). The fully adjusted model demonstrated an association between the C-index (per unit increase) and future diabetes risk across AIP levels. This association strengthened as the AIP levels increased, particularly at higher levels, suggesting that elevated AIP levels may reflect a higher metabolic burden, enhancing its modulatory effect on the C-index. This aligns with the stratified analysis results, where higher AIP levels consistently amplified the effect of the C-index on the risk of diabetes. As the control variables increased, the independent effect of the C-index gradually weakened across all AIP strata, suggesting that its association with diabetes risk was influenced by other factors, particularly fatty liver and HbA1c, which were included as additional control variables in Model 3. These findings underscore the importance of considering metabolic context and key confounders when evaluating the C-index. To further elucidate these mechanisms, future research should focus on exploring the mediating roles of fatty liver, HbA1c, and AIP in the association between the C-index and diabetes risk.

### Mediating effect of AIP, fatty liver, and HbA1c on C-index and diabetes risk

**Fig. 6:**
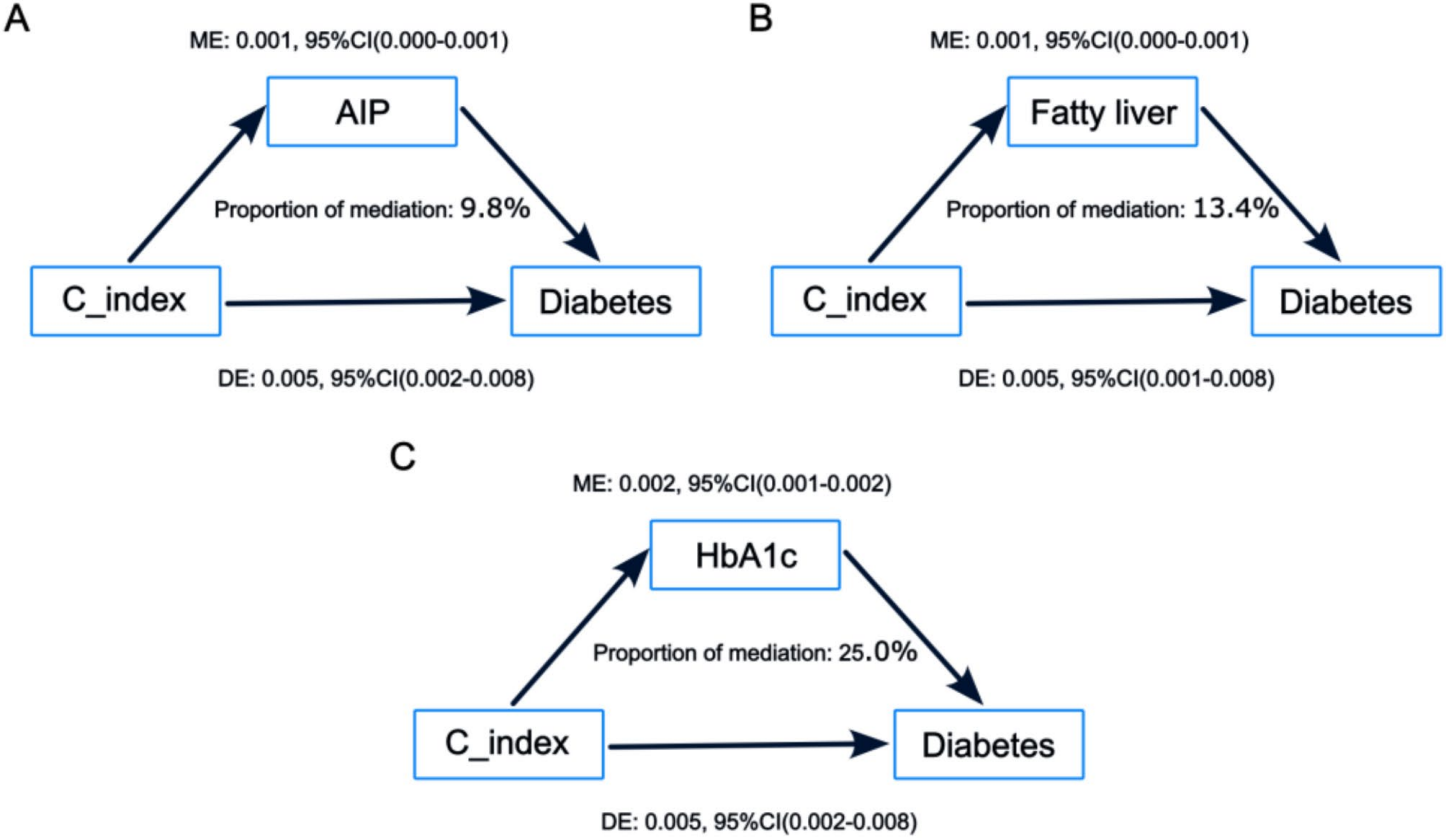
Mediation analysis for C-index and incident diabetes via AIP, fatty liver, and HbA1c

We further conducted a mediation analysis, which revealed that AIP, fatty liver, and HbA1c all played significant mediating roles in the association between the C-index and diabetes risk (Fig. 6, Supplementary Table S3). Specifically, AIP accounted for 9.8% of the mediation effect, whereas HbA1c and fatty liver accounted for 25.0% and 13.4%, respectively, highlighting their distinct roles in modulating the risk of diabetes. These findings suggest that HbA1c, with its highest mediation proportion, may serve as a key metabolic regulator in this pathway, whereas fatty livers may contribute through other complementary mechanisms. Importantly, statistical significance was confirmed for all mediation effects (*p* < 0.05).

### Enhanced diabetes risk prediction with C-index model integration of AIP, fatty liver, and HbA1c

**Fig. 7:**
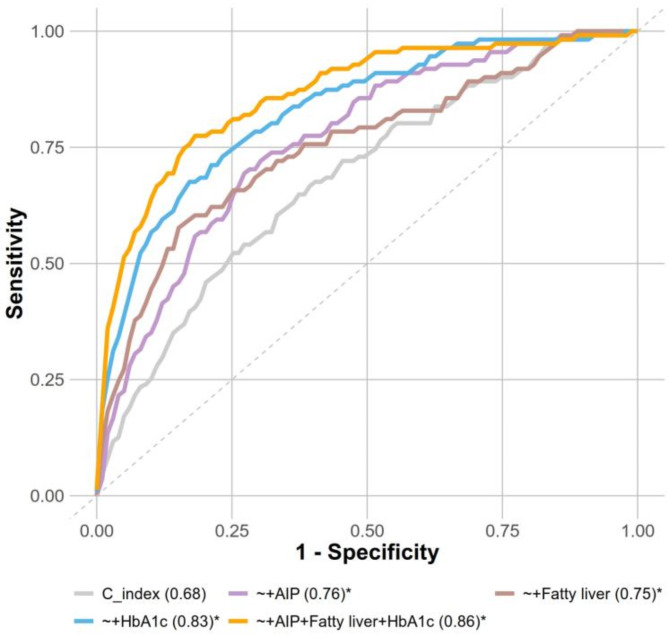
ROC curve for a model combining AIP, fatty liver, and HbA1c to predict incident diabetes

The addition of AIP to the C-index model significantly improved the predictive accuracy for incident diabetes, as indicated by the higher AUC value (0.76) compared to the C-index model alone (AUC = 0.68) (Fig. 7). Similarly, the incorporating fatty liver (AUC = 0.75) or HbA1c (AUC = 0.83) into the C-index model further enhanced the predictive performance. Notably, the combined model, integrating AIP, fatty liver, and HbA1c levels with the C-index, achieved the highest predictive performance (AUC = 0.86). Statistically significant differences in AUC between the combined models and the standalone C-index were confirmed by DeLong’s test (*p* < 0.05). However, no significant multiplicative interactions were observed between C-index and AIP (HR = 0.91, 95% CI 0.78–1.05), Fatty liver (HR = 1.14, 95% CI 0.90–1.43), or HbA1c (HR = 1.05, 95% CI 0.94–1.16) in relation to incident diabetes risk. These findings demonstrate a stepwise improvement in diabetes risk prediction as additional independent metabolic factors, such as AIP, fatty liver, and HbA1c, were incorporated. This led in the optimal performance of the combined model, highlighting the importance of integrating multiple independent metabolic indicators for a more robust and precise assessment of diabetes risk.

## Discussion

Limited research exists regarding C-index-AIP interactions, with the majority of investigations focusing on individuals exhibiting prediabetic characteristics. This study initially investigates potential C-index-AIP interactions for future diabetes events in a normoglycemic individual cohort, which constitutes the largest dataset in the current analysis, comprising approximately 15,453 normoglycemic participants. We observed that elevated AIP levels were independently associated with increased diabetes risk in normoglycemic individules, consistent with their established roles as markers of dyslipidemia and adiposity [26]. These associations were diminished after adjusting for confounding factors, suggesting that their predictive value may partially depend on other metabolic variables. Notably, a high AIP level (≥ 1.21) remained significantly associated with a 27% increase in diabetes risk, even after full adjustment, underscoring its potential as a robust marker for metabolic risk stratification. Moderate to high Cumulative AIP exposure not only increases the risk of diabetes progression but also hinders its regression within a certain range [27]. 

Moreover, we reiterates the well-established that elevated levels of C-index wre independently associated with increased diabetes risk [28]. The quartile grouping of the C-index in the study showed that higher C-index levels were significantly associated with an increased risk of diabetes, which is consistent with the findings of a Chinese cohort study [22]. As adjustment variables were progressively added, this relationship became less apparent in the normoglycemic population, possibly due to population characteristics. Our RCS analysis revealed a dose-response relationship between C-index and diabetes risk as AIP levels increased, suggesting that higher AIP levels may amplify the effect of the C-index on diabetes risk by increasing metabolic burden, thereby exacerbating the susceptibility conferred by adiposity [29]. The stratified analysis by AIP further confirmed this result, especially in the range of AIP 0.11–1.21, where the association between the C-index and diabetes risk was attenuated but still present after adjusting for key confounders such as fatty liver and HbA1c. The AIP > 1.21 group showed a marginally significant association. Regardless of whether AIP was categorized using various cutoff points or treated as a continuous variable, this association disappeared or weakened after adjusting for key confounders, such as fatty liver and HbA1c, suggesting that these factors may mediate or confound the relationship. Thus, the influence of the C-index on diabetes risk may be modified by baseline metabolic risk [30], with AIP, fatty liver, and HbA1c playing key roles in mediating these effects. The predictive value of the C-index depends on the underlying lipid and metabolic profiles, which provides a critical context for its influence on diabetes development.

AIP, fatty liver, and HbA1c, indicators of lipid and glucose metabolism, influence the relationship between the C-index and diabetes risk through different mechanisms. This investigation represents the first to examine the intermediary role of AIP, fatty liver, and HbA1c in C-index and diabetes risk. Mediation analysis found that AIP and fatty liver accounted for 9.8% and 13.4%, respectively, of the mediation effect in the relationship between the C-index and diabetes risk, whereas HbA1c demonstrated more substantial mediating effects, accounting for 25.0%. An elevated C-index indicates the accumulation of abdominal and visceral fat, which is often accompanied by an increase in serum free fatty acid (FFAs) levels. Excessive lipid accumulation in the liver leads to fatty liver, promoting the release of excess serum FFAs into the bloodstream [31]. These FFAs are converted into triglycerides via the glycerol-3-phosphate pathway. This results in the accumulation of various lipid metabolites, which in turn induce insulin resistance [32]. This plays a role in the relationship between the C-index and diabetes risk, promoting the development of diabetes. Simultaneously, an increase in triglyceride levels may lead to elevated AIP levels, which further exacerbates lipid metabolism abnormalities, intensifying the role of the C-index in diabetes risk. Additionally, fatty liver may exacerbate metabolic dysregulation by releasing inflammatory cytokines, further increasing the risk of diabetes. [33–34] As insulin resistance worsens, the effectiveness of insulin decreases, leading to poor blood sugar control and onset of hyperglycemia. Elevated blood sugar (reflected by increased HbA1c levels) further exacerbates lipid metabolism disorders, creating a vicious cycle in which hyperglycemia promotes the accumulation of fatty acids, leading to fatty liver disease and insulin resistance, [35] which in turn elevates blood sugar levels to remain elevated, ultimately resulting in the development of diabetes. Therefore, AIP, fatty liver, and HbA1c exacerbating lipid metabolism abnormalities and insulin resistance influence the relationship between the C-index and diabetes risk, thereby promoting the development of diabetes.

The present study assessed the mediating role of AIP, fatty liver, and HbA1c in the association between the C-index and diabetes risk and further ROC analyses showed that AIP, fatty liver, and HbA1c significantly improved not only in predicting incident diabetes but also in mediating C-index-related diabetes risk. Our research demonstrated a higher AUC (0.68 for the C-index model in predicting diabetes, which is slightly higher than the AUC of 0.59 reported in previous studies [28]. However, the integration of AIP, fatty liver, and HbA1c into the C-index model significantly improved predictive performance, with the combined model achieving the highest AUC (0.86), outperforming the diabetes risk prediction model that includes 11 factors (AUC: 0.73) [35]. This underscores the importance of incorporating multiple metabolic indicators for a robust risk prediction. Clinicians should consider using combined models in normoglycemic populations to identify high-risk individuals early, and tailor prevention strategies accordingly.

This study elucidates the intricate interactions among biomarkers such as AIP, fatty liver, and HbA1c with diabetes risk in normoglycemic individuals. These findings provide valuable insights for diabetes screening and prevention strategies. Specifically, individuals who are overweight or obese, particularly those with abdominal obesity, should monitor these indicators vigilantly. The strengths of this study include its large sample size, rigorous statistical adjustments, and the use of mediation and stratified analyses to clarify complex relationships. Nevertheless, the observational design of this study limits causal inference. Exposures and outcomes were not randomly assigned, potentially leading to undetected significant confounders that may affect the accuracy of mediation analyses, and residual confounding cannot be entirely excluded. Consequently, the homogeneity of the normoglycemic cohort may reduce generalizability. Future research should explore the molecular mechanisms linking fatty liver, HbA1c, AIP, C-index, and diabetes risk, validate these findings in more diverse populations, and incorporate novel biomarkers, such as genetic and proteomic indicators, to enhance the predictive accuracy and deepen the understanding of diabetes pathogenesis.

## Conclusion

This study elucidates the potential of the AIP and C-index as significant predictors of diabetes risk in normoglycemic populations. The interrelationship between these biomarkers and their interactions with metabolic mediators, such as fatty liver and HbA1c, underscores the necessity for a multifaceted approach to predict diabetes risk. By integrating multiple metabolic indicators, clinicians can achieve more precise and personalized risk stratification, facilitating targeted preventive strategies. Subsequent investigations should explore the molecular mechanisms underlying the relationships among fatty liver, HbA1c, AIP, C-index, and diabetes risk, with particular emphasis on validating the interactions of these biomarkers across diverse populations and disease contexts to elucidate the underlying mechanisms.

## Electronic supplementary material

Below is the link to the electronic supplementary material.

Supplementary Material 1

## Data Availability

Dataset is publicly accessible via the Dryad repository (https://datadryad.org/stash/dataset/doi:10.5061/dryad.8q0p192).
