# Supplementary material for: AIP, fatty liver, and HbA1c as modifiers of the C-index and diabetes risk relationship
Source: Lipids Health Dis. 2025 Apr 2;24:129. doi: 10.1186/s12944-025-02546-1 (PMC11963646; doi:10.1186/s12944-025-02546-1)
Supplement: Supplementary file 1 — Supplementary Material 1 [file 12944_2025_2546_MOESM1_ESM.docx]

Supplementary Table S1 Association Between AIP or C_index and Risk of Diabetes in Normoglycemia Individuals at Baseline

|  | **Model 1** | | **Model 2** | | **Model 3** | |
| --- | --- | --- | --- | --- | --- | --- |
|  | **HR (95% CI)** | **p-value** | **HR (95% CI)** | **p-value** | **HR (95% CI)** | **p-value** |
| **AIP** |  |  |  |  |  |  |
| Per SD increase | 1.61 (1.43-1.82) | <0.001 | 1.41 (1.23-1.59) | <0.001 | 1.31 (1.15-1.48) | <0.001 |
| AIP <0.11 | REF |  | REF |  | REF |  |
| AIP ≥0.11 - <1.21 | 1.60 (1.18 - 2.18) | 0.003 | 1.30(0.94-1.78) | 0.108 | 1.19(0.87-1.62) | 0.277 |
| AIP ≥1.21 | 2.93 (2.06 - 4.16) | <0.001 | 1.95 (1.36-2.81) | <0.001 | 1.58(1.11-2.26) | 0.011 |
| Ordinal scale | 1.74 (1.46-2.06) | <0.001 | 1.42 (1.19-1.70) | <0.001 | 1.27(1.07-1.52) | 0.006 |
| **C_index** |  |  |  |  |  |  |
| Per SD increase | 1.81 ( 1.61-2.03) | <0.001 | 1.51(1.35-1.71) | <0.001 | 1.29 (1.14-1.45) | <0.001 |
| Fourths |  |  |  |  |  |  |
| 1 | REF |  | REF |  | REF |  |
| 2 | 1.28 (0.83- 1.98) | 0.257 | 1.20 (0.77-1.85) | 0.418 | 1.01 (0.65 - 1.57) | 0.951 |
| 3 | 1.19 (0.78- 1.82) | 0.421 | 1.03 (0.67-1.59) | 0.901 | 0.90 (0.58 - 1.38) | 0.62 |
| 4 | 1.88 (1.25- 2.84) | 0.002 | 1.48 (0.97-2.25) | 0.068 | 1.35 (0.89- 2.05) | 0.153 |
| Ordinal scale | 1.24(1.11-1.40) | <0.001 | 1.14 (1.02-1.29) | 0.027 | 1.15 (1.02-1.30) | 0.023 |

Cox regression risk and the data are presented as Hazard ratios (HRs) with their 95% confidence interval (95% CI). REF. = reference.

Model 1: Adjusted for age, sex, systolic pressure,exercise habits, smoking status, drinking status, ALT, AST, GGT,AIP.

Model 2: additionally adjusted for fatty liver.

Model 3: additionally adjusted for HbA1c.

Supplementary Table S2 Association Between C_index Levels and Future Diabetes Events, according to AIP Index

| **A)** | **AIP <0.11** | | **AIP ≥ 0.11** | |
| --- | --- | --- | --- | --- |
|  | **HR (95% CI)** | **p-value** | **HR (95% CI)** | **p-value** |
| **Model 1** |  |  |  |  |
| Continuous (per SD) | 1.43(1.11-1.87) | 0.006 | 1.43(1.27-1.62) | <0.001 |
| Categorized (fourths) |  |  |  |  |
| C_index 1 | REF. |  | REF. |  |
| 2 | 1.19(0.45 - 3.09) | 0.728 | 0.92(0.61 - 1.39) | 0.687 |
| 3 | 1.52(0.61 - 3.77) | 0.367 | 1.27(0.86 - 1.88) | 0.223 |
| 4 | 2.20(0.93 - 5.2) | 0.072 | 1.82(1.26 - 2.64) | 0.002 |
| **Model 2** |  |  |  |  |
| Continuous (per SD) | 1.31(1.002-1.72) | 0.049 | 1.28(1.13-1.45) | <0.001 |
| Categorized (fourths) |  |  |  |  |
| C_index 1 | REF. |  | REF. |  |
| 2 | 1.18(0.45 - 3.06) | 0.741 | 0.79(0.52 - 1.20) | 0.275 |
| 3 | 1.32(0.53 - 3.31) | 0.552 | 1.03(0.70 - 1.54) | 0.868 |
| 4 | 1.79(0.74 - 4.32) | 0.195 | 1.33(0.91 - 1.94) | 0.142 |
| **Model 3** |  |  |  |  |
| Continuous (per SD) | 1.27(0.97-1.67) | 0.077 | 1.28(1.13-1.44) | <0.001 |
| Categorized (fourths) |  |  |  |  |
| C_index 1 | REF. |  | REF. |  |
| 2 | 1.02(0.39 - 2.67) | 0.967 | 0.74(0.48 - 1.13) | 0.162 |
| 3 | 1.11(0.43 - 2.85) | 0.832 | 0.97(0.66 - 1.44) | 0.896 |
| 4 | 1.57(0.64 - 3.85) | 0.322 | 1.36(0.94 - 1.97) | 0.104 |

| **B)** | **AIP < 0.11** | | **AIP ≥ 0.11- ≤ 1.21** | | **AIP > 1.21** | |
| --- | --- | --- | --- | --- | --- | --- |
|  | **HR (95% CI)** | **p-value** | **HR (95% CI)** | **p-value** | **HR (95% CI)** | **p-value** |
| **Model 1** |  |  |  |  |  |  |
| Continuous (per SD) | 1.43(1.11-1.87) | 0.006 | 1.53(1.31 - 1.79) | <0.001 | 1.2(1.01 - 1.43) | 0.042 |
| Categorized (fourths) |  |  |  |  |  |  |
| C_index 1 | REF. |  | REF. |  | REF. |  |
| 2 | 1.19(0.45 - 3.09) | 0.728 | 1.20(0.71 - 2.04) | 0.500 | 1.21(0.68 - 2.16) | 0.520 |
| 3 | 1.52(0.61 - 3.77) | 0.367 | 1.31(0.78 - 2.2) | 0.305 | 1.44(0.82 - 2.53) | 0.201 |
| 4 | 2.20(0.93 - 5.2) | 0.072 | 2.22(1.37 - 3.59) | 0.001 | 1.66(0.97 - 2.86) | 0.067 |
| **Model 2** |  |  |  |  |  |  |
| Continuous (per SD) | 1.31(1.002-1.72) | 0.049 | 1.17(1.31 - 1.62) | <0.001 | 1.12(0.94 - 1.33) | 0.214 |
| Categorized (fourths) |  |  |  |  |  |  |
| C_index 1 | REF. |  | REF. |  | REF. |  |
| 2 | 1.18(0.45 - 3.06) | 0.741 | 1.06(0.62 - 1.81) | 0.836 | 1.17(0.66 - 2.09) | 0.592 |
| 3 | 1.32(0.53 - 3.31) | 0.552 | 1.091(0.64 - 1.84) | 0.756 | 1.30(0.74 - 2.28) | 0.363 |
| 4 | 1.79(0.74 - 4.32) | 0.195 | 1.65(1.008 -2.70) | 0.046 | 1.40(0.81 - 2.42) | 0.222 |
| **Model 3** |  |  |  |  |  |  |
| Continuous (per SD) | 1.27(0.97-1.67) | 0.077 | 1.40(1.19-1.64) | < 0.001 | 1.12(0.94 - 1.33) | 0.405 |
| Categorized (fourths) |  |  |  |  |  |  |
| C_index 1 | REF. |  | REF. |  | REF. |  |
| 2 | 1.02(0.39 - 2.67) | 0.967 | 1.07(0.62 - 1.84) | 0.806 | 1.28(0.71 - 2.3) | 0.405 |
| 3 | 1.11(0.43 - 2.85) | 0.832 | 1.07(0.63 - 1.81) | 0.805 | 1.35(0.76 - 2.39) | 0.309 |
| 4 | 1.57(0.64 - 3.85) | 0.322 | 1.70(1.05 - 2.76) | 0.030 | 1.65(0.96 - 2.83) | 0.071 |

Cox regression risk and the data are presented as Hazard ratios (HRs) with their 95% confidence interval (95% CI). REF. = reference.

Model 1: Adjusted for age, sex, systolic pressure,exercise habits, smoking status, drinking status, ALT, AST, GGT.

Model 2: additionally adjusted for fatty liver.

Model 3: additionally adjusted for HbA1c.

Supplementary Table S3 Mediation analysis for C_index Levels and incident diabetes via AIP, fatty liver and HbA1c

| Mediator | Total effect | Mediation effect | Direct effect | PM(%) | p-value of PM |
| --- | --- | --- | --- | --- | --- |
| AIP | 0.005(0.002-0.008) | 0.001(0.000-0.001) | 0.005(0.002-0.008) | 9.8 | <0.001 |
| Fatty Liver | 0.005(0.002-0.008) | 0.001(0.000-0.001) | 0.005(0.002-0.008) | 13.4 | <0.001 |
| HbA1c | 0.005(0.002-0.008) | 0.001(0.000-0.001) | 0.005(0.002-0.008) | 25.0 | <0.001 |
